# C-Reactive Protein as a Screening Test for Tuberculosis in People Living with HIV in Southern India: A Cross-Sectional, Observational Study

**DOI:** 10.3390/jcm11133566

**Published:** 2022-06-21

**Authors:** Aishwarya Saripalli, John Ramapuram

**Affiliations:** Department of Medicine, Kasturba Medical College, Mangalore 575001, India; john@ramapuram.net

**Keywords:** C reactive protein, tuberculosis, HIV, screening test

## Abstract

Background: Tuberculosis is the leading cause of mortality in people living with HIV(PLHIV). We assessed the utility of C-reactive protein (CRP) as a screening test for tuberculosis (TB) in PLHIV. Methods: We performed a cross-sectional, observational study on 150 HIV patients visiting the Anti-Retroviral Therapy (ART) center for the follow up of their ART treatment. Patients who screened positive on the WHO symptom screen were included in the study. C-reactive protein levels in the blood were measured, and the patients were followed up with for a confirmatory diagnosis of tuberculosis. Results: The ideal cut-off for CRP was found to be 8.25. There was a statistically significant relationship between the CRP value and tuberculosis positivity (*p* value < 0.001). The CRP value had a sensitivity of 70.13%, a specificity of 69.86%, a positive predictive value of 71.05%, a negative predictive value of 68.92%, and a total diagnostic accuracy of 70% in patients who screened positive on the WHO symptom screen. Conclusion: CRP is a valuable screening tool and should be added to the tuberculosis screening algorithm to improve the diagnostic accuracy of screening for tuberculosis in people living with HIV.

## 1. Introduction

Tuberculosis affects HIV-positive individuals throughout all phases of HIV infection and is the leading cause of mortality among HIV positive patients [1]. Tuberculosis and human immunodeficiency virus/acquired immunodeficiency syndrome (HIV/AIDS) constitute a significant percent of infectious disease in resource-limited countries.

Globally, an estimated 10.0 million (range, 8.9–11.0 million) [2] people fell ill with tuberculosis in 2019, a number that has been declining very slowly in recent years. There were an estimated 1.2 million (range, 1.1–1.3 million) deaths due to tuberculosis among HIV-negative individuals in 2019 (a reduction from 1.7 million in 2000) and an additional 208,000 deaths (range, 177,000–242,000) [3] among HIV-positive patients (a reduction from 678,000 in 2000).

Currently, the standard screening procedures include using a four-part symptom screen alone or in conjunction with chest radiography (CXR) for people living with HIV (PLHIV) in high-burden settings [1]. PLHIV who report any of the symptoms of current cough, fever, weight loss, or night sweats may have active tuberculosis; they should be evaluated for tuberculosis and other diseases and should be offered preventive treatment if active tuberculosis is excluded.

The WHO’s target product profile for a tuberculosis screening test requires that the sensitivity be ≥90% and the specificity be ≥70% [4]. Current tools to screen people living with HIV for active pulmonary tuberculosis are limited to symptom-based screening, which has an unacceptably low specificity, and chest X-ray, which has a relatively low sensitivity. Chest X-ray-based screening has high infrastructural requirements and relies on trained interpreters, both of which are not routinely available in most health care centers in tuberculosis-endemic areas [5].

C-reactive protein (CRP) is a non-specific inflammatory marker that has been found to be elevated in both HIV-infected and uninfected people with pyogenic infections including active tuberculosis [6]. CRP is an acute phase reactant, the levels of which rise in response to IL-6 mediated pyogenic infections such as active tuberculosis [7]. CRP has been proposed to be a potential biomarker for tuberculosis disease as well as a prognostic indicator of disease and treatment [8]. CRP testing has the advantage of being quick and inexpensive. Although elevations in CRP (≥10 mg/L) are not specific for active tuberculosis, two studies that evaluated CRP as a screening test among PLHIV initiating antiretroviral therapy (ART) found that CRP has a two- to six-fold higher specificity (58% and 81%) than symptom-based tuberculosis screening [9,10]. However, previous studies have only looked at CRP as a screening tool for pulmonary tuberculosis and have failed to assess its utility in the screening of extra pulmonary tuberculosis.

This study was conducted to determine the diagnostic accuracy of CRP for the detection of tuberculosis in people living with HIV who are positive according to the WHO symptom screen. It evaluated the utility of CRP as a screening tool for pulmonary as well as extra-pulmonary tuberculosis.

## 2. Materials and Methods

### 2.1. Aim

The aim of this study was to determine the diagnostic accuracy of C-reactive protein for the detection of tuberculosis in people living with HIV who are positive according to the WHO symptom screen.

### 2.2. Objectives

1. To estimate the sensitivity and specificity of C-reactive protein as a screening test for tuberculosis in people living with HIV who are positive according to the WHO symptom screen.
2. To calculate the ideal CRP cut-off value to be used in TB screening in PLHIV who are positive according to the WHO symptom screen.

### 2.3. Study Design and Setting

This was a cross-sectional, observational study that was conducted in the Anti-Retroviral Therapy (ART) centers of tertiary care hospitals attached to Kasturba Medical College, Mangalore, a city in southern India. Data collection was conducted between January 2020 and May 2021. The study was conducted following approval from the institutional ethics committee of Kasturba Medical College, Mangalore.

### 2.4. Sampling

The sample size was calculated using the ‘*n* Master sample size calculator’ based on a CRP sensitivity of 89% [11], precision of 5%, and a confidence interval of 95%. The sample size was found to be 150. Samples were collected from January 2020 to May 2021.

Patients diagnosed as HIV positive, who come to the Anti-Retroviral Therapy center for follow up, those of who were positive according to the WHO symptom screen, were considered for the study. All the study participants were over 18 years of age and provided written informed consent for their participation in the study. Sampling was convenience based. All consecutive eligible patients were enrolled until the sample size was met.

Patients with HIV who had been initiated on Anti-Tuberculosis Therapy (ATT) for any diagnosis of tuberculosis were excluded. Patients with any other auto-immune disease were also excluded.

### 2.5. Study Description

From January 2020 to May 2021, patients presenting to the Anti-Retroviral Therapy center for follow-up treatment and who qualified as per the inclusion and exclusion criteria were enrolled in the study. Demographic data and other relevant data with respect to patient’s HIV treatment and immunological status were documented. Blood samples from all the patients who were positive according to the WHO symptom screen were sent for C-reactive protein estimation. A value of ≥10 mg/L (rounding to the nearest whole number) was considered as being positive on the CRP screening test (this value was selected based on previous studies [5,7]). All patients were subsequently followed up with for a diagnosis of tuberculosis by a physician. The diagnosis of pulmonary TB was made through a nucleic acid amplification test (GeneXpert) using a sputum specimen or bronchoalveolar lavage fluid. The diagnosis of extra pulmonary TB was made based on the site of tuberculosis. Tubercular pleural effusion was diagnosed when pleural fluid analysis revealed an exudative effusion with ADA > 40 U/L or the presence of a positive GeneXpert for Mycobacterium tuberculosis. A diagnosis of tubercular lymphadenitis was made when either a fine-needle aspiration specimen or a lymph node biopsy specimen showed a histopathological confirmation of tuberculosis or was positive on GeneXpert. A diagnosis of abdominal tuberculosis was made when a tissue biopsy specimen or ultrasound-guided aspirate was positive on GeneXpert or was confirmed to be tuberculosis according to histopathology. The objectives of the study were two-fold. First, the objective was to estimate the sensitivity and specificity of C-reactive protein as a screening test for tuberculosis in people living with HIV. Additionally, the second aim of the study was to calculate the ideal CRP cut-off value to be used in tuberculosis screening in PLHIV.

### 2.6. Statistical Methods

Tuberculosis-positive was considered as the primary outcome variable. The CRP value was considered as the primary explanatory variable. Descriptive analysis was carried out according to the mean, standard deviation, or median for quantitative variables and by frequency and proportion for categorical variables. Data were also represented using appropriate diagrams such as bar graphs, pie charts and stacked bar graphs. The categorical outcomes were compared between study groups using the Chi square test.

CRP-positive was considered as the screening test, and tuberculosis-positive was considered as the gold standard. The utility of the CRP value in predicting tuberculosis positivity was assessed by means of receiver operative curve (ROC) analysis. The area under the ROC curve, along with its 95% CI and *p* value, is presented in the results. Based on the ROC analysis, it was decided that 8.25 would be considered the ideal cut-off value. The sensitivity, specificity, predictive values, and diagnostic accuracy of the screening test along with their 95% CI are presented in the results. A *p* value of < 0.05 was considered statistically significant. Data were analyzed using coGuide software, V.1.03 (producer—coGuide, headquarters—Bengaluru, India).

## 3. Results

From January 2020 to May 2021, a total of 150 samples were collected, and all 150 samples were included in the final analysis.

### 3.1. Demographic Data

**Figure 1:**
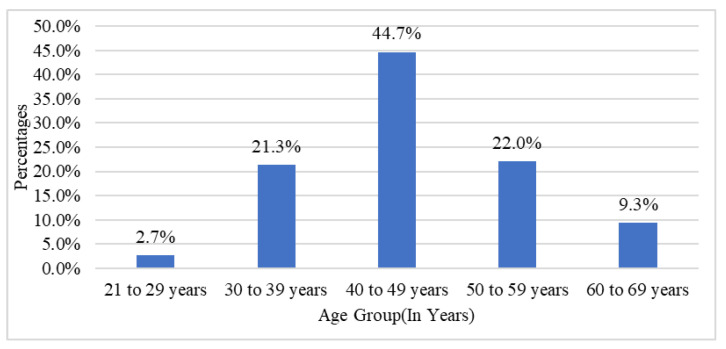
Bar graph of age groups in the study population.

The mean age of the study participants was 46.16 ± 9.22 years old and ranged from 21 to 68 years. Among the study population, most of the patients were aged between 40 and 49 years, i.e., 67 (44.67%) (Figure 1). Among the study population, 83 (55.33%) participants were male, and 67 (44.67%) participants were female.

In the study population, the mean CD4 count was found to be 308.91 ± 84.38, ranging from 176 to 600, with a median value of 289. Among the viral load, 21 (14%) had an undetectable viral load (Target Not Detected-TND) (Table 1). Out of 150 patients, the following ART regimens were used: 135 (90%) were on TLE (Tenofovir, Lamivudine, Efavirenz), 9 (6%) were on ZLN (Zidovudine, Lamivudine, Nevirapine), 3 (2%) were on TL-AR (Tenofovir, Lamivudine, boosted PI- Atazanavir/Ritonavir), and 3 (2%) were on TLD (Tenofovir, Lamivudine, Dolutegravir).

The median CRP value among the study participants was 8.40 and ranged from 0.50 to 46.23. Out of 150 participants, 67 (44.67%) were found to be CRP-positive, and a total of 77 (51.33%) were tuberculosis-positive. Among those found to have tuberculosis, 58 (75.32%) participants had pulmonary tuberculosis, eight (10.38%) had tubercular lymphadenitis, seven (9.09%) had tubercular pleural effusion, and four (5.19%) had abdominal tuberculosis (Table 2). The data regarding different modalities used to diagnose extra-pulmonary TB are presented in Table 3. Table 4 represents the median CRP values among different types of TB patients.

Out of the 77 participants with tuberculosis, CRP was found to be positive in 50 (64.94%) individuals and negative for 27 (35.06%). Out of the 73 participants without tuberculosis, the CRP value was positive for 17 (23.29%) of them and negative for 56 (76.71%). We found that there was a statistically significant relationship between CRP and tuberculosis positivity (*p* value < 0.001) (Table 5). CRP had a sensitivity of 64.94% (95% CI 53.22% to 75.47%) in predicting the presence of tuberculosis, a specificity of 76.71% (95 CI 65.35% to 85.81%), a false-positive rate of 23.29% (95 CI 14.19% to 34.65%), and a false-negative rate of 35.06% (95 CI 24.53% to 46.78%). The positive predictive value was found to be 74.63% (95 CI 62.51% to 84.47%), and the negative predictive value was 67.47% (95 CI 56.30% to 77.35%). The total diagnostic accuracy was 70.67% (95 CI 62.69% to 77.81%) (Table 6).

### 3.2. Receptor Operator Curve Analysis

**Figure 2:**
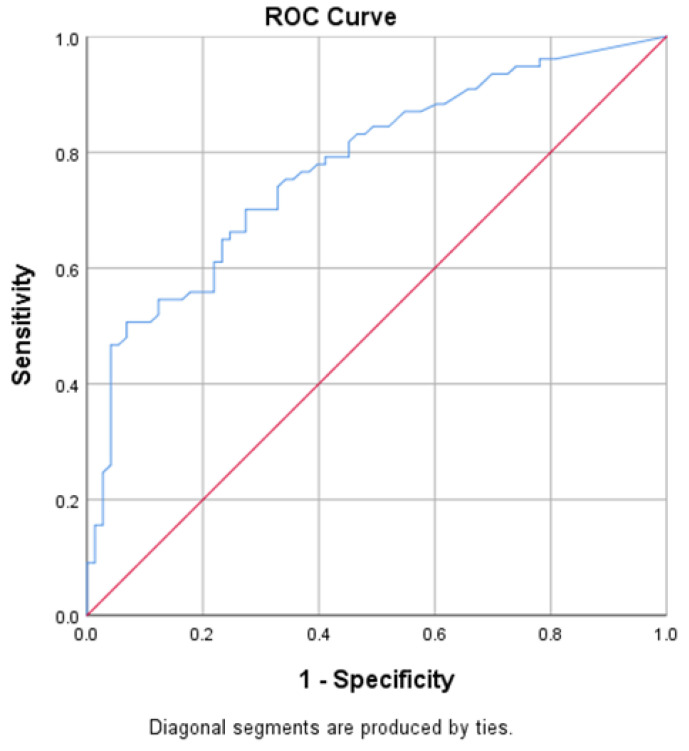
Predictive validity of the CRP value in predicting TB positivity.

CRP had fair validity in predicting tuberculosis, as indicated by an area under the curve of 0.777 (95% CI 0.703 to 0.851, *p* value < 0.001) (Figure 2).

### 3.3. Ideal CRP Value

The ROC curve and data presented in Table 7 indicate that the screening test has a good predictive value. From the ROC analysis, the ideal cut-off for CRP was found to be 8.25 mg/L. Using this cut-off, CRP was found to be high (≥8.25) for 54 (70.13%) participants and low (<8.24) for 23 (29.87%) participants. Out of 73 participants without tuberculosis, the CRP value was high (≥8.25) for 22 (30.14%) participants and low (<8.24) for 51 (69.86%) participants. There was a statistically significant relationship between the ideal CRP value and tuberculosis positivity (*p* value < 0.001) (Table 8). Table 9 represents the percentage positivity of CRP among different types of TB, considering the ideal CRP value as the cut-off. The ideal CRP value had a sensitivity of 70.13% (95% CI 58.62% to 80.03%) in predicting tuberculosis, a specificity of 69.86% (95 CI 58% to 80.06%), a false-positive rate of 30.14% (95 CI 19.94% to 42%), and a false-negative rate of 29.87% (95 CI 19.97% to 41.38%). The positive predictive value of CRP was found to be 71.05% (95 CI 59.51% to 80.89%), and the negative predictive value was 68.92% (95 CI 57.10% to 79.17%), with a total diagnostic accuracy of 70% (95 CI 61.99% to 77.20%) (Table 10).

## 4. Discussion

Tuberculosis and HIV are public health problems that have a synergistic effect on each other. In people living with HIV (PLHIV), tuberculosis increases HIV replication [6], while HIV lowers the immune response against tuberculosis, leading to increased active tuberculosis infection, re-infection, or reactivation, thus increasing the risk of progression from latent tuberculosis to active tuberculosis. CRP is a non-specific marker of inflammation. Although CRP values have been found to be elevated in HIV-positive individuals both before and after the initiation of ART without underlying tuberculosis, studies have shown that the CRP value is usually <3 mg/dL, irrespective of their immune status (i.e., CD4 count and viral load) [12,13].

In studies by Shapiro AE et al. [8] and Lawn SD et al. [9], it was found that CRP values were significantly elevated in those with tuberculosis compared to those without tuberculosis. Previous studies have only looked at CRP as a screening tool for the detection of pulmonary tuberculosis in people living with HIV. Ours is the first to have studied the utility of CRP as a screening tool for both pulmonary as well as extra pulmonary tuberculosis. The four-part WHO symptom screen had a sensitivity of 79%, a specificity of 50%, and a negative predictive value of 97.7% at a tuberculosis prevalence of 5%, as reported in a meta-analysis by the WHO [14].

In this study, after ROC analysis of the data, we found that a CRP value of 8.25 mg/dL which provided optimum sensitivity and specificity, had fair predictive validity in detecting tuberculosis, as indicated by an area under the curve value of 0.777 (95% CI 0.703 to 0.851, *p* value < 0.001). CRP (≥8.25 mg/dL) had a sensitivity of 70.13% and a specificity of 69.86% in predicting tuberculosis, and the total diagnostic accuracy was 70% in the present study.

The results of this study were comparable with the diagnostic accuracy reported by other studies. There was variation in reporting the diagnostic accuracy of CRP with regard to the gold standard used (Culture/ Gene Xpert MTB-CB NAAT/ Sputum positive) because other studies included pulmonary tuberculosis only, in which it is easier to obtain the microbiological evidence of tuberculosis that is necessary for its diagnosis. Because our study included extra pulmonary tuberculosis, it also accepted other modalities to reach a diagnosis of tuberculosis, such as obtaining microbiological evidence in some situations, for example in cases of tubercular pleural effusion, abdominal tuberculosis, etc., may not have been practical.

In the study by Shapiro AE et al. [8], using CRP to discriminate between the presence of tuberculosis (positive culture) and the absence of tuberculosis (negative culture) resulted in an area under the ROC curve of 0.80. Using a CRP threshold >5 mg/L resulted in a sensitivity of 90.5% and a specificity of 58.5%. Using a CRP threshold >10 mg/L, the sensitivity decreased to 78.6%, and the specificity increased to 72.3%. Lawn SD et al. [9] observed that very low (<1.5 mg/L) CRP concentrations excluded tuberculosis (100% negative predictive value), whereas very high concentrations (>400 mg/L) were strongly predictive of tuberculosis (100% positive predictive value). The area under the curve (AUC) was 0.81 when all patients were included and was similar when the analysis was restricted to patients with a positive WHO symptom screen. Yoon C et al. [5] observed that compared to POC CRP, the WHO symptom screen had higher sensitivity (89% vs. 96%) but substantially lower specificity (72% vs. 14%, *p* < 0·0001). They observed that POC CRP met the minimum TB screening test sensitivity (≥90%) and specificity (≥70%) targets when the cut-point was lowered to 8 mg/L (AUROC 0·80, 95% CI: 0·77 to 0·83) or 9 mg/L (AUROC 0·81, 95% CI: 0·78 to 0·83.

From this study, it was concluded that the relationship between CRP-positive (with a cut-off value of 10 mg/L) and tuberculosis was found to be statistically significant. CRP was found to have to a sensitivity of 64.94% and a specificity of 76.71%. After ROC analysis, the ideal cut-off for CRP was found to be 8.25 (with a sensitivity of 70.13% and a specificity of 69.86%).

The current WHO-consolidated guidelines on tuberculosis include CRP as a screening tool for tuberculosis in treatment-naïve individuals with HIV as a conditional recommendation. The cut-off value recommended by the WHO is >5 mg/L [15]. Our study helps to reiterate the value of CRP as a screening tool for TB in PLHIV and shows its validity in patients who are on anti-retroviral therapy as well as its utility in extra-pulmonary tuberculosis. The ideal cut-off value as per our study was chosen to optimize the sensitivity and specificity. Our data suggest that a cut-off value of >5 mg/L would result in a sensitivity and specificity of 81% and 54%, respectively.

The limitations of this study were that the sample size was small, thus reducing the power of the study. The study did not use TB cultures as the gold standard, which may have resulted in missed cases. Previous studies have shown that the sensitivity and specificity of GeneXpert was found to be 88.5% and 96.7% for the detection of pulmonary TB and 85.1% and 95.7% for the detection of extra-pulmonary TB [16]. The diagnostic gold standard used to detect extra-pulmonary TB has its challenges. It does not provide microbiological evidence of tuberculosis and involves invasive procedure which may not be feasible in all patients. Although in some cases of tuberculosis, obtaining tissue/microbiological evidence of TB maybe challenging, and in practice, patients maybe be treated based on a clinical diagnosis of TB, we avoided using this as a criterion for diagnosis to maintain objectivity. This study used lab CRP values for screening and not-point-of care-CRP, which may be more appropriate for screening.

## 5. Conclusions

From our study, the relationship between being CRP-positive (with a cut-off value of 10 mg/L) and tuberculosis was found to be statistically significant. CRP was found to have to a sensitivity of 64.94% and a specificity of 76.71%. After ROC analysis, the ideal cut-off for CRP was found to be 8.25 (with a sensitivity of 70.13% and a specificity of 69.86%).

CRP is a valuable screening tool and should be added to the tuberculosis screening algorithm to improve diagnostic accuracy of screening for tuberculosis in people living with HIV.

## Figures and Tables

**Table 1:** Descriptive analysis of viral load in the study population.

Viral Load	Frequency	Percentages
Target Not Detected	21	14%
Data unavailable	129	86%

**Table 2:** Descriptive analysis of type of TB in the study population.

Type of Tuberculosis	Frequency	Percentages
Pulmonary TB	58	75.32%
Tubercular lymphadenitis	8	10.38%
Tubercular pleural effusion	7	9.09%
Abdominal TB	4	5.19%

**Table 3:** Percentage of extra-pulmonary Tuberculosis diagnosed by different modalities.

Diagnostic Modality (*n* = Total Samples Tested)	Total Number Positive	Percentage Positive
GeneXpert (*n* = 19)	4	21.05%
ADA (*n* = 7)	6	85.71%
Histopathology (*n* = 12)	9	75%

**Table 4:** Median C-Reactive protein values for different types of TB.

Tuberculosis	Median CRP Value
Pulmonary TB	14.65
Tubercular lymphadenitis	3.75
Tubercular pleural effusion	13.2
Abdominal TB	6
TB negative	4.3

**Table 5:** Comparison of CRP positivity and TB.

C-Reactive Protein	Tuberculosis		Chi Square	*p* Value
	Positive (*n* = 77)	Negative (*n* = 73)		
Positive (*n* = 67)	50 (64.94%)	17 (23.29%)	26.298	<0.001

**Table 6:** Validity of C-Reactive Protein in predicting Tuberculosis (with a CRP cut-off value of 10 mg/L).

Parameter	Value	95% Confidence Interval	
		Lower	Upper
Sensitivity	64.94%	53.22%	75.47%
Specificity	76.71%	65.35%	85.81%
False positive rate	23.29%	14.19%	34.65%
False negative rate	35.06%	24.53%	46.78%
Positive predictive value	74.63%	62.51%	84.47%
Negative predictive value	67.47%	56.30%	77.35%
Diagnostic accuracy	70.67%	62.69%	77.81%

**Table 7:** Value of area under the curve for test variable CRP.

Test Result Variable(s): CRP Value				
Area Under the Curve	Std. Error	95% Confidence Interval of AUC		*p* Value
		Lower Bound	Upper Bound	
0.777	0.038	0.703	0.851	<0.001

**Table 8:** Comparison between ideal C-Reactive Protein values and Tuberculosis.

C-Reactive Protein	Tuberculosis		Chi Square	*p* Value
	Positive (*n* = 77)	Negative (*n* = 73)		
High (≥8.25)	54 (70.13%)	22 (30.14%)	23.979	<0.001
Low (<8.24)	23 (29.87%)	51 (69.86%)		

**Table 9:** Ideal C-Reactive Protein value (≥8.25) and types of TB.

Type of Tuberculosis	CRP Positive	CRP Negative
Pulmonary TB	47 (81.03%)	11 (18.97%)
Tubercular lymphadenitis	1 (12.5%)	7 (87.5%)
Tubercular pleural effusion	5 (71.42%)	2 (28.85%)
Abdominal TB	1 (25%)	3 (75%)

**Table 10:** Validity of ideal C-Reactive Protein value in predicting tuberculosis.

Parameter	Value	95% Confidence Interval	
		Lower	Upper
Sensitivity	70.13%	58.62%	80.03%
Specificity	69.86%	58.00%	80.06%
False positive rate	30.14%	19.94%	42.00%
False negative rate	29.87%	19.97%	41.38%
Positive predictive value	71.05%	59.51%	80.89%
Negative predictive value	68.92%	57.10%	79.17%
Diagnostic accuracy	70.00%	61.99%	77.20%

## Data Availability

Study data are available in the Supplementary Materials.

## Supplementary Materials

The following supporting information can be downloaded at: https://www.mdpi.com/article/10.3390/jcm11133566/s1, Include study data.

## Abbreviations

HIV	Human immunodeficiency virus
PLHIV	People living with HIV
CRP	C-reactive protein
TB	Tuberculosis
WHO	World Health Organization
AIDS	Acquired immunodeficiency syndrome
CXR	Chest X-Ray
ART	Anti-retroviral therapy
ROC	Receiver operator curve
CI	Confidence interval
SD	Standard deviation
TND	Target not detected
TLE	Tenofovir + Lamivudine + Efavirenz
ZLN	Zidovudine + Lamivudine + Nevirapine
TL-AR	Tenofovir + Lamivudine + Atazanavir (Ritonavir-boosted)
TLD	Tenofovir + Lamivudine + Dolutegravir
CBNAAT	Cartridge based nucleic acid amplification test
AUROC	Area under receiver operator curve
ADA	Adenosine deaminase
